# Artificial Intelligence Revolutionizing Plastic Surgery Scientific Publications

**DOI:** 10.7759/cureus.40770

**Published:** 2023-06-21

**Authors:** Mohd Altaf Mir

**Affiliations:** 1 Burns and Plastic Surgery, All India Institute of Medical Sciences, Bathinda, Bathinda, IND

**Keywords:** aesthetic surgery, data curation, scientific publications, plastic surgery, artificial intelligence

## Abstract

The integration of AI in plastic surgery scientific publications is revolutionizing the field by enabling efficient data analysis, improving surgical planning, predicting outcomes, and facilitating evidence-based advancements. The ongoing collaboration between AI researchers and plastic surgeons promises to enhance patient care, increase surgical precision, and drive innovation in plastic surgery practice.

## Editorial

AI has been transforming various industries, and the field of plastic surgery is no exception. With its potential to enhance precision, efficiency, and accuracy, AI is revolutionizing the way plastic surgery publications are conducted. This ground-breaking technology has the power to revolutionize the field, improving patient outcomes and providing valuable insights to surgeons and researchers. As AI continues to advance, it holds immense promise for the future of plastic surgery publications [1-4]. 

One of the significant contributions of AI to plastic surgery publications is its ability to enhance precision and accuracy. AI algorithms can analyze vast amounts of data, including medical images, patient records, and surgical outcomes. By utilizing this wealth of information, AI systems can identify patterns, predict potential complications, and even suggest optimal treatment plans.

For instance, AI-powered imaging analysis can assist surgeons in evaluating the potential aesthetic outcomes of various procedures. By analyzing past surgical cases and incorporating factors such as facial symmetry, anatomical proportions, and cultural preferences, AI algorithms can generate visual simulations to guide surgeons in making informed decisions. This not only improves surgical precision but also allows patients to have a clearer understanding of the expected results [1-3].

AI also plays a crucial role in improving the efficiency of plastic surgery publications. Traditionally, compiling research and analyzing data can be a time-consuming process. However, AI algorithms can automate these tasks, reducing the burden on researchers and accelerating the pace of publications. By analyzing a vast amount of data in a fraction of the time it would take a human, AI systems can help identify trends, uncover new insights, and support evidence-based decision-making.

Moreover, AI-powered chatbots and virtual assistants can streamline the dissemination of information. These intelligent systems can respond to inquiries, provide personalized recommendations, and guide surgeons and researchers through the vast pool of published literature. By quickly retrieving relevant articles and summarizing key findings, AI can significantly improve the efficiency of accessing information, ultimately enhancing the quality and timeliness of plastic surgery publications [2-4].

Moreover, there are opposing viewpoints on the use of artificial intelligence in plastic surgery which can be summarized as follows.

1. Some individuals argue that relying heavily on AI in plastic surgery may undermine the importance of skilled human surgeons. They believe that AI should only be used as an assistive tool rather than a replacement for surgeons' expertise and decision-making abilities. They express concerns that excessive reliance on AI algorithms might lead to a decline in the quality of patient care and the overall patient-doctor relationship.

2. Opponents of AI in plastic surgery raise ethical concerns regarding privacy, consent, and potential biases embedded within AI algorithms. They argue that AI systems may not adequately consider cultural or individual preferences, leading to standardized beauty ideals that perpetuate societal biases and discrimination. Additionally, they worry that the use of AI in patient data analysis might compromise privacy and data security.

3. Critics of AI in plastic surgery emphasize the need for transparency and accountability in AI algorithms. They argue that the proprietary nature of AI systems used in surgical decision-making can hinder transparency, making it difficult for both surgeons and patients to understand the basis of AI-generated recommendations. This lack of transparency raises questions about accountability in case of errors or adverse outcomes.

4. Opponents express concerns about the potential dehumanization of healthcare when AI is extensively utilized. They worry that excessive reliance on AI algorithms may lead to a loss of empathy and patient-centered care. They argue that human interaction, communication, and the ability to understand patients' emotions and concerns are crucial aspects of plastic surgery that cannot be fully replaced by AI.

5. Critics highlight the challenges associated with training AI algorithms in plastic surgery due to limited and biased datasets. They argue that the lack of diverse representation in training data could lead to algorithmic biases, making the AI systems less accurate or reliable. Additionally, opponents express concerns about the generalization of AI algorithms to diverse patient populations, as the training data may not adequately capture the full range of variations and complexities seen in real-world plastic surgery cases.

By adopting the below proactive solutions, the ethical concerns surrounding AI in plastic surgery can be effectively addressed, promoting responsible and patient-centered use of AI in the field.

1. AI systems in plastic surgery must adhere to strict ethical guidelines to prevent plagiarism. Proactive measures should include:

a. AI systems should be equipped with algorithms that can compare the generated outcomes with a vast database of existing procedures, ensuring originality.

b. AI-generated outcomes should be clearly identified as such and credit given to the human developers or surgeons involved in training the AI model.

c. Independent audits should be conducted to evaluate AI systems for potential plagiarism and ensure compliance with ethical standards.

2. Addressing the potential for errors in AI systems used in plastic surgery requires a proactive approach:

a. AI models should be trained using diverse and representative datasets, including different body types, ethnicities, and ages. Regular validation and retraining of the models can help reduce errors and improve accuracy.

b. AI should be used as a tool to support human surgeons rather than replace them entirely. Expert surgeons should be actively involved in decision-making and exercise their judgment when assessing AI-generated recommendations.

c. Patients should be fully informed about the limitations and potential errors associated with AI systems.

3. Transparency regarding the AI's role in the decision-making process is crucial for obtaining informed consent.

4. Recognizing and addressing the emotional human concerns related to AI in plastic surgery is essential:

a. Surgeons should foster open and honest communication with patients, emphasizing the collaborative nature of the decision-making process. Empathy, active listening, and addressing emotional concerns can help build trust.

b. AI should be used to enhance the overall patient experience rather than replace human interaction. Ensuring that patients feel heard, valued, and understood is crucial in plastic surgery, where emotional factors often play a significant role.

c. Plastic surgery organizations and professional bodies should develop and enforce ethical guidelines for the integration of AI in the field. These guidelines should emphasize the importance of considering emotional factors and preserving the doctor-patient relationship.

As the use of AI in plastic surgery publications expands, it is essential to address ethical considerations. Transparent guidelines and stringent regulations should be established to ensure that AI systems are reliable and unbiased and respect patient privacy. The development and implementation of standardized protocols will be crucial to validate AI algorithms, assess their accuracy, and mitigate potential risks.

Furthermore, collaboration between AI developers, plastic surgeons, and researchers is crucial to maintain ethical standards. Together, they can establish guidelines on the appropriate use of AI in plastic surgery publications, ensuring that patient safety and well-being remain paramount [4,5].

AI is ushering in a new era for plastic surgery publications. By harnessing the power of AI, the field can achieve higher precision, greater efficiency, and improved patient outcomes. The ability of AI algorithms to analyze complex datasets, generate simulations, and streamline information retrieval will undoubtedly transform how plastic surgeons and researchers approach their work. However, it is imperative that this technology is implemented ethically and responsibly to maintain patient trust and uphold the highest standards of care. As AI continues to advance, the future of plastic surgery publications holds tremendous promise, shaping the field for the better and revolutionizing the way we approach aesthetic medicine.

Summarizing that AI has made significant contributions to the field of plastic surgery, revolutionizing various aspects of patient care and surgical procedures. Here are some key roles of AI in plastic surgery:

1. AI algorithms can analyze patient data, such as medical images and clinical records, to assist surgeons in planning and simulating procedures. This technology enables surgeons to visualize potential outcomes, assess different treatment options, and optimize surgical plans for better results.

2. AI-based image analysis tools can accurately analyze medical images, such as preoperative scans or photographs, to identify anatomical structures, measure dimensions, and detect abnormalities. This aids in precise diagnosis, treatment planning, and tracking patient progress over time.

3. Virtual reality (VR) and augmented reality (AR) technologies combined with AI algorithms allow surgeons to immerse themselves in a virtual surgical environment. This enables them to practice complex procedures, improve their skills, and explore different surgical techniques without posing risks to actual patients.

4. AI-powered robotic systems, such as the da Vinci Surgical System, have been increasingly utilized in plastic surgery. These systems enhance surgical precision, dexterity, and control, enabling surgeons to perform intricate procedures with minimal invasiveness and improved patient outcomes.

5. AI algorithms can assist in automating the identification and analysis of patterns and features relevant to plastic surgery, such as skin lesions, facial symmetry, or scar assessment. This helps in the early detection of conditions, accurate measurement, and objective evaluation of treatment efficacy.

6. By analyzing large datasets and patient profiles, AI can provide personalized treatment recommendations based on individual characteristics, medical history, and desired outcomes. This assists surgeons in tailoring procedures to each patient's unique needs, optimizing results, and minimizing risks.

7. AI algorithms can help monitor patients' progress post-surgery, analyzing data from wearable devices or medical sensors. This real-time monitoring aids in the early detection of complications, prompt intervention, and personalized postoperative care.

8. AI facilitates the analysis of vast amounts of patient data, electronic health records, and scientific literature. It can identify patterns, correlations, and insights that may have otherwise been overlooked. This supports evidence-based decision-making, advancements in surgical techniques, and improved patient safety.

While AI continues to advance in plastic surgery, it is important to note that its role complements and assists surgeons rather than replacing them. Human expertise, clinical judgment, and patient-doctor communication remain crucial in delivering optimal outcomes and maintaining patient safety.
